# Molecular detection of genes encoding resistance to tetracycline and determination of plasmid-mediated resistance to quinolones in avian pathogenic *Escherichia coli* in Sukabumi, Indonesia

**DOI:** 10.14202/vetworld.2018.1581-1586

**Published:** 2018-11-18

**Authors:** Ryan Septa Kurnia, Agustin Indrawati, Ni Luh Putu Ika Mayasari, Adin Priadi

**Affiliations:** 1 Department of Animal Disease and Veterinary Health, Faculty of Veterinary Medicine, Bogor Agricultural University (IPB), Indonesia; 2 Animal Health Diagnostic Unit, PT Medika Satwa Laboratories, West Java, Indonesia

**Keywords:** Antibiotic, colibacillosis, *Escherichia coli*, genes, resistance

## Abstract

**Aim:**

This study aimed to identify genes encoding resistance to tetracycline (TE) and plasmid-mediated resistance to quinolones in *Escherichia coli* isolates from clinical cases of avian colibacillosis in Sukabumi, Indonesia.

**Materials and Methods:**

A total of 25 *E. coli* archive isolates were collected in 2013-2017 from clinical cases of avian colibacillosis in Sukabumi, Indonesia. All isolates were tested for TE and quinolone resistance using the disk diffusion method. TE -resistant *E. coli* isolates were screened for the presence of *tet(A)* and *tet(B)* genes by single polymerase chain reaction (PCR). The *qnr*(*A*), *qnr*(*B*), and *qnr*(*S*) genes were detected by multiplex PCR in quinolone-resistant *E. coli* isolates.

**Results:**

Result of this study shows that 19 of 25 (76%) *E. coli* isolates are resistant to oxytetracycline and 64% are resistant to TE; among them, 63.2% and 31.5% were positive *tet(A)* and *tet(B)*, respectively. 13 out of 25 (52%) are resistant to ciprofloxacin and 36% are resistant to enrofloxacin either norfloxacin; among them, 61.6% were positive *qnr*(*A*), 7.7% were positive *qnr*(*B*), 23% were positive *qnr*(*S*), and 7.7% were positive both of *qnr*(*A*) and *qnr*(*S*).

**Conclusion:**

This study shows that a few pathogens of *E. coli* are resistant to TE and quinolone. The frequency of *tet* and *qnr* genes that are responsible for this resistance among avian pathogenic *E. coli* isolates in Sukabumi, Indonesia, was high.

## Introduction

Avian colibacillosis is one of the most important infectious diseases in birds of all ages. This disease has an important economic impact on poultry production worldwide. Antibiotic as feed additives in an animal is required to reduce the economic consequences of bacterial disease, but it was contributed to the spread of antibiotic-resistant bacteria [1-3]. Susceptible bacteria become resistant to an antibiotic through genetic mutation or through horizontal transfer. Antibiotic exposure is a source of stress that can create stress-induced resistance among bacteria [4].

Tetracycline (TE) is one of the oldest antibiotics that is used in the livestock industry. Over the years, TE has been improved and marketed under various trade names. Importantly, they are also used as growth promoters of farm animals worldwide. It is not surprising that resistance to these antibiotics has spread in various bacterial communities [5]. The resistance mechanisms for the TE class of antibiotics fall in four categories such as energy-dependent efflux pumps, ribosomal protection proteins, enzymatic inactivation, and target modification are recognized as mediators of TE resistance in bacteria. The genes associated with an efflux mechanism, namely *tet*(*A*), *tet*(*B*), *tet*(*C*), *tet*(*D*), and *tet*(*E*), are important part of TE resistance in *E. coli* [6]. Other antibiotic agents that used to treat infections caused by this bacterium include fluoroquinolones, newer quinolones containing a fluorine atom. In veterinary medicine, a large proportion of these antimicrobials belongs to a group of drugs with the so-called restricted indication [7]. Quinolone usage is threatened by the rising occurrence of resistance, which has been observed in every species that is treated by this drug class. Recent work has helped to define how quinolones interact with gyrase or topoisomerase IV and how mutations in can lead these enzymes to resistance [8]. Recent studies have shown that the emergence of plasmid-determined quinolone resistance may contribute by several means to the rapid increase in bacterial resistance to quinolones. Plasmid-mediated quinolone resistance genes *qnr*(*A*), *qnr*(*B*), and *qnr*(*S*) encode pentapeptide repeat protein and has ability to protect DNA gyrase from quinolone inhibition [9].

Horizontal transfer of antibiotics resistance genes has also been confirmed, which is mediated by plasmids. Plasmid-mediated resistance widespread in poultry among Enterobacteriaceae isolates and the effect of their combination with other resistance mechanisms have been little studied in Indonesia. The aim of this study was to investigate the presence of *tet* and *qnr* genes among *Escherichia coli* isolates from clinical cases of avian colibacillosis in Sukabumi, Indonesia.

## Materials and Methods

### Ethical approval

Samples were collected from the case of colibacillosis at Sukabumi area farm from 2013 to 2017. Archival is a collection of PT Medika Satwa Laboratories, West Java, Indonesia. No live animals were used in the present study. Therefore, ethical approval was not required in this study.

### Bacterial isolates and biochemical characterization

A total of 25 non-duplicated archive isolates avian pathogenic *E. coli* (APEC) were collected from the case of colibacillosis at Sukabumi area farm from 2013 to 2017. Archival is a collection of PT Medika Satwa Laboratories, West Java, Indonesia, and *E. coli* ATCC 25922 as reference isolate. Isolates were conventionally inoculated on MacConkey agar and Triple Sugar Iron Agar and then incubated at 37°C. Samples also inoculated in *E. coli* broth with MUG (EC-MUG broth) at 37°C for 22 h. In the presence of MUG, *E. coli* produces the enzyme glucuronidase that hydrolyzes MUG to yield a fluorogenic product that is detectable under UV light (366 nm). Congo red dye agar test (CR test) used to differentiate invasive and non-invasive *E. coli* in poultry. The colonies were streaked on CR agar and incubated for 72 h at 25°C. The identification of *E. coli* was based on the results of diagnostic tests, which included Gram staining, colonial morphology, gas production, and the ability to be enriched in the EC-MUG broth and compared to the standard *E. coli* strain, ATCC 25922 [10-12]. Identified *E. coli* isolate cultured on blood agar for a further step of research.

### Antibiotic sensitivity identification

A total of 25 *E. coli* isolates were screened for TE and fluoroquinolone resistance using the disk diffusion method according to the Clinical and Laboratory Institute (CLSI) guidelines [13]. A bacterial suspension with an optical density equal to 0.5 McFarland standard (*ca*.1.5×10^8^ CFU/ml) was inoculated onto Mueller-Hinton agar for disk diffusion testing. The following antibiotic disks were used for antimicrobial susceptibility testing: TE 30 µg, oxytetracycline (OT) 30 µg, ciprofloxacin (CIP) 5 µg, enrofloxacin (ENR) 5 µg, and norfloxacin (NOR) 10 µg. All the disks were placed on the plate and then incubated aerobically for 18-24 h at 35°C. The reference strain *E. coli* ATCC 25922 was used as a control. The zone diameters for all antibiotics were measured and interpreted as susceptible, intermediate, or resistance according to the criteria recommended by the Laboratory Standards Institute (CLSI) and manufacturer protocols (Oxoid, UK).

### Preparation of DNA template

Bacterial colonies from overnight blood agar at 37°C were picked using sterile pipette tips and aseptically suspended in 500 µL of sterile distilled water in microtubes; the suspensions were boiled for 10 min. After centrifugation for 5 min at 16 060× *g*, 100 µL of the supernatants were taken as DNA templates for the polymerase chain reaction (PCR) reaction [11].

### Resistance gene detection

Molecular detection by PCR and multiplex PCR assay was used to evaluate the presence of *tet*(*A*), *tet*(*B*), *qnr*(*A*), *qnr*(*B*), and *qnr*(*S*) resistance genes in TE - and/or quinolone-resistant strains of APEC. Single PCR reactions were performed in 10 µL reactions containing DNA template, primers (Table-1) [14], and PCR kit (KAPA2G Fast Hotstart Readymix, Wilmington, USA). The amplification condition was 3 min at 95°C and 30 cycles each consisting of 95°C for 1 min, annealing temperature (Table-1) for 30 s and 72°C for 1 min, followed by a final extension step of 5 min at 72°C. Amplified samples were analyzed by electrophoresis in 1.5% agarose gel and stained by ethidium bromide. A molecular weight marker with 100 bp (VC 100 bp Plus DNA Ladder Vivantis, Selangor, Malaysia) was used as a standard size. Multiplex PCR reactions were performed in 10 µL reactions containing DNA template, primers (Table-2) [9], and PCR kit (KAPA2G Fast Multiplex PCR Kit, Wilmington, USA). The amplification condition was 3 minutes at 95°C and 30 cycles each consisting of 95°C for 1 min, annealing temperature (Table-2) for 30 s and 72°C for 1 min, followed by a final extension step of 5 min at 72°C. Amplified samples were analyzed by electrophoresis in 1.5% agarose gel and stained by ethidium bromide. A molecular weight marker with 100 bp (VC 100 bp Plus DNA Ladder Vivantis, Selangor, Malaysia) was used as a standard size.

**Table-1:** *E. coli*-resistant genes and primer sequences used for single PCR identification.

Antibiotics	Resistance gene	Primer sequence	Size (bp)	Annealing temperature	Reference
Tetracycline	*tet* *(A)*	(F) 5′-GGTTCACTCGAACGACGTCA-3′	577	57°C	[14]
		(R) 5′-CTGTCCGACAAGTTGCATGA-3′			
	*tet (B)*	(F) 5′-CCTCAGCTTCTCAACGCGTG-3′	634	56°C	[14]
		(R) 5′-GCACCTTGCTCATGACTCTT-3′			

PCR=Polymerase chain reaction, *E. coli=Escherichia coli*

**Table-2:** *E. coli*-resistant genes and primer sequences used for multiplex PCR identification.

Antibiotics	Resistance gene	Primer sequence	Size (bp)	Annealing temperature	Reference
Quinolone	*qnr(A)*	(F) 5′-ATTTCTCACGCCAGGATTTG-3′	516	55°C	[9]
		(R) 5′-GATCGGCAAAGGTTAGGTCA-3′			
	*qnr(B)*	(F) 5′-GATCGTGAAAGCCAGAAAGG-3′	469	55°C	[9]
		(R) 5′-ACGATGCCTGGTAGTTGTCC-3′			
	*qnr(S)*	(F) 5′-ACGACATT CGTCAACTGCAA-3′	417	55°C	[9]
		(R) 5′-TAAATTGGCACCCTGTAGGC-3′			

PCR=Polymerase chain reaction, *E. coli=Escherichia coli*

## Results

### Bacterial isolates and biochemical characterization

All archive isolates APEC strains (25 isolates) showed pink colonies on MacConkey agar which is typical for *E. coli* and rod-shaped cell in Gram staining. In addition, biochemical characterization shows that all the strains were capable of producing a fluorogenic product, which is a typical characteristic of E. coli. Inoculation in CR test shows that all the strain positive reaction for CR test with binding dye on the colony.

### Antibiotic sensitivity identification

A total 22 out of 25 (88%) APEC isolates were resistant to at least one of the tested antibiotics based on inhibition zone by disk diffusion methods (Table-3). The result showed that APEC isolates from clinical cases of avian colibacillosis in Sukabumi 76% (19 of 25 isolates) are resistant to oxytetracycline and 64% (16 out of 25 isolates) are resistant to TE. Among them, 8% (2 of 25 isolates) were intermediate to TE (Table-4). Resistance to quinolones is also known to be 52% (13 of 25 isolates) resistant to CIP and 36% (9 of 25 isolates) resistant to ENR and NOR. Among them, 8% (2 of 25 isolates), 16% (4 out of 25 isolates), and 4% (1 out of 25 isolates) were intermediate to CIP, ENR, and NOR, respectively.

**Table-3:** Antibiotic resistance pattern of avian pathogenic *E. coli* isolates.

No.	Isolates	TE^a^	OT^b^	CIP^c^	EN^d^	NOR^e^
1	MSL E.C 1	R	R	R	R	I
2	MSL E.C 2	R	R	S	S	S
3	MSL E.C 3	R	R	I	I	S
4	MSL E.C 4	R	R	R	S	R
5	MSL E.C 5	I	R	S	S	S
6	MSL E.C 6	S	S	S	S	S
7	MSL E.C 7	S	S	S	S	S
8	MSL E.C 9	S	S	R	R	R
9	MSL E.C 10	R	R	R	R	R
10	MSL E.C 11	S	R	S	S	S
11	MSL E.C 14	R	R	R	S	S
12	MSL E.C 15	S	S	R	I	S
13	MSL E.C 16	R	R	R	R	R
14	MSL E.C 17	R	R	S	S	S
15	MSL E.C 18	S	S	R	R	R
16	MSL E.C 19	S	S	S	S	S
17	MSL E.C 20	R	R	S	S	S
18	MSL E.C 28	R	R	R	R	R
19	MSL E.C 29	R	R	R	R	R
20	MSL E.C 30	R	R	R	R	R
21	MSL E.C 31	I	R	R	R	R
22	MSL E.C 32	R	R	I	I	S
23	MSL E.C 33	R	R	R	I	S
24	MSL E.C 34	R	R	S	S	S
25	MSL E.C 35	R	R	S	S	S

a TE=Tetracycline,

b OT=Oxytetracycline,

c CIP=Ciprofloxacin,

d EN=Enrofloxacin,

e NOR=Norfloxacin.

E. coli: Escherichia coli

**Table-4:** Number and percentage of resistant APEC strains against antibiotics.

Antibiotic class	Antibiotics	Isolates *E. coli* (n=25)		

		Sensitive (%)	Intermediate (%)	Resistant (%)
Tetracycline	TE	28	8	64
	OT	24	0	76
Quinolone	CIP	40	8	52
	ENR	48	16	36
	NOR	60	4	36

TE=Tetracycline, OT=Oxytetracycline, CIP=Ciprofloxacin, EN=Enrofloxacin, NOR=Norfloxacin. *E. coli=Escherichia coli,* APEC=Avian pathogenic *Escherichia coli*

### Resistance gene amplification

**Figure-1:**
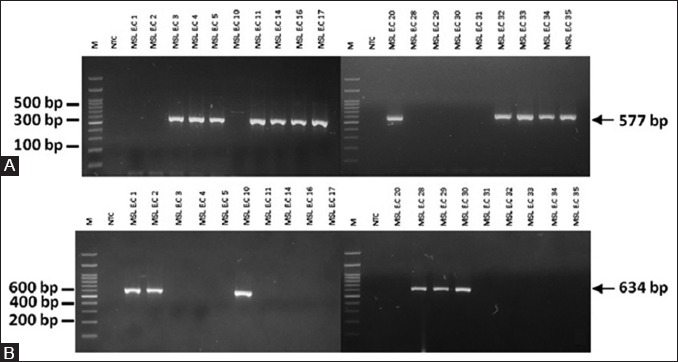
Amplification of *tet(A)* and *tet(B)* gene determinants on agarose gel. (A) An approximate 577 bp band size represents *tet(A)*. M: 100 bp DNA ladder. NTC: Non-template control. (B) An approximate 634 bp band size represents *tet(B)*. M: 100 bp DNA ladder. NTC: Non-template control.

Single PCR result shows that 19 of TE-resistant isolates, 63.2% (12 out of 19 isolates) were *tet*(*A*) positive and 31.6% (6 out of 19 isolates) were *tet*(*B*) positive (Figure-1). One of 19 TE -resistant isolates (MSL E.C 31) were *tet*(*A*) and *tet*(*B*) negative. Multiplex PCR result shows that 13 of quinolone-resistant, 61.6% (8 out of 13 isolates) were *qnr*(*A*) positive, 7.7% (1 out of 13 isolates) were *qnr*(*B*) positive, and 23% (3 out of 13 isolates) were *qnr*(*S*) positive. One of 13 (7.7%) quinolone-resistant isolates (MSL E.C 15) were *qnr*(*A*) and *qnr*(*S*) positive. The result of sensitivity and gene detection indicated that all resistant isolates harbor one or more antibiotic resistance genes (Tables-4 and 5).

**Table-5:** Molecular characterization of antimicrobial resistance genotypes among APEC isolates.

Antibiotic class	Number of resistance isolates (n=25)	Associated genes tested	Number of positive isolates (%)
Tetracycline	19 (76%)	*tet (A)*	12 (63.2)
		*tet (B)*	6 (31.6)
Quinolone	13 (52%)	*qnr (A)*	8 (61.6)
		*qnr (B)*	1 (7.7)
		*qnr (S)*	3 (23)
		*qnr (A)* and *qnr (S)*	1 (7.7)

APEC=Avian pathogenic *Escherichia coli*

## Discussion

Avian colibacillosis is an infectious disease of birds caused by *E. coli*, which is considered as one of the principal causes of morbidity and mortality, associated with heavy economic losses to the poultry industry by its association with various disease conditions [1]. Antibiotics treatment is often required to improve animal welfare and reduce the economic consequences of the disease. The most commonly administered antibiotic treatments include amoxicillin, OT, or ENR [2]. Antibiotic exposure is a source of stress that can create stress-induced resistance among bacteria as part of the SOS response. Under the response, mutations and genetic exchanges occur inside bacterial DNA. As a consequence of these genetic manipulations, any gene sequence, including those for antibiotic targets, can be altered, providing the possibility for the evolution of resistance [3].

In this study, the resistance rates to TE and OT were 64% and 76%, indicating a high rate of resistance. TE is frequently used as growth promoters of farm animals worldwide. Over the years, TE antibiotics have been improved and marketed under various trade names. Salehi and Bonab investigated 50 APEC strains isolated from broilers with colisepticemia and found that the rate of resistance to OT was 95% and TE was 94% [15]. The relationship between the use of TE as a growth promoter in the poultry industry and the development of resistance has been established by many authors during the past decades. Such long-term subtherapeutic use of TE antibiotics in feed is of great concern because it leads to selective pressure on the *E. coli* carried by the poultry and also in the farms environment, so the beneficial effects of TE will become limited as bacterial resistance increases [6]. Detection of the respective genes encoding resistance to TE raises a possibility to analyze epidemiological aspects of the resistance and to discover mobile genetic elements that may have contributed in the dissemination of the TE resistance genes (*tet*) in nature [5]. This gene associated with an efflux mechanism, namely *tet*(*A*), *tet*(*B*), *tet*(*C)*, *tet*(*D*), and *tet*(*E*), is an important part of TE resistance in *E. coli* [16]. Molecular detection of *tet(A)* and *tet(B)* genes of 19 TE resistant isolates in this study shows 63.2% *tet(A)* positive and 31.3% *tet(B)* positive. This study also found one isolate (MSL E.C 31) which is negative to *tet*(*A*) or *tet*(*B*). Possibly other *tet* genes are involved in the resistance to TE in the isolates MSL E.C 31, among other *tet*(*C*), *tet*(*D*), and *tet*(*E*). These results supported the observation that these *tet*(*A*) and *tet*(*B*) efflux genes are the most frequent *tet* genes found in Enterobacteriaceae. High prevalence of these genes variant might be associated with horizontal gene transfer by conjugative or transposons that implicated in the efflux mechanism that leads to resistance phenotype of bacteria [17]. Van *et al*. [18] examined a current resistance profile of *E. coli* from poultry in Vietnam. They collected feces from two poultry farms where chickens were <1 month old. They reported that the *tet*(*A*) gene was the most prevalent of the TE resistance genes (71.1% of the isolates), followed by *tet*(*B*) (18.4%) in Vietnam. Sengelov *et al*. [19] found that the *tet*(*A*) and *tet*(*B*) were the most prevalent than *tet*(*C*), *tet*(*D*), or *tet*(*E*) in Denmark. The majority of isolates contained *tet*(*A*) and *tet*(*B*) which is in agreement with Al-Bahry *et al*. [20] findings that most of *tet* determinant is associated with either conjugative or mobilized elements, which explain their wide distribution among bacteria.

**Figure-2:**
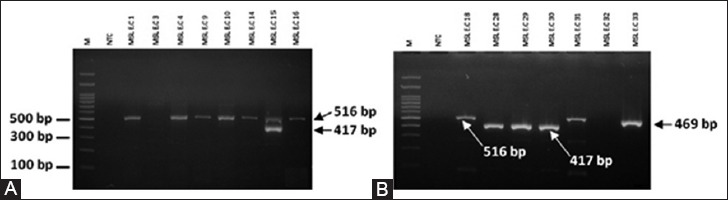
Multiplex polymerase chain reaction (PCR) amplification *qnr(A)*. *qnr(B)*, and *qnr(S)* on agarose gel. (A) M: 100 bp DNA ladder. NTC: Non-template control. PCR product MSL E.C 15 represents both *qnr (A)* and *qnr(S)* genes in this study. (B) M: 100 bp DNA ladder. NTC: Non-template control.

The resistance rates of our samples to quinolone antibiotics shows that, 52% isolates were CIP resistant and 36% isolates were ENR and NOR resistant. The World Health Organization (WHO) has classified these drugs as critically important in human medicine and said that efforts to reduce antibiotic use in farm animals should priorities the fluoroquinolones and another antibiotic class known as the modern [21]. Under current legislation, if a small number of chickens show signs of ill-health, ENR can be added to the drinking water of the whole flock for up to 10 days at a time, even when most of the birds are not ill. A recent report from The European Medicines Agency sets out statistics which show that fluoroquinolone use as oral solutions is higher than the treatment of individual animals [22]. Yeh *et al*. [23] reported 50.6% ENR resistance and 42.3% CIP resistance of *E. coli* isolates from poultry in Taiwan. The resistance of quinolone in European countries in poultry was about 52% [7]. The main mechanism of quinolone resistance is the accumulation of mutations in the bacterial enzymes targeted by fluoroquinolones to DNA gyrase and DNA topoisomerase IV. Plasmid-mediated horizontally transferable gene encoding quinolone resistance has shed light on these phenomena. Qnr proteins are capable of protecting DNA gyrase from quinolones and have been circulated for at least 20 years [24]. In this study, *qnr*(*A*), *qnr*(*B*), and *qnr*(*S*) genes were detected in APEC isolates within 61.6% *qnr*(*A*) positive, 7.7% *qnr*(*B*) positive, and 23% *qnr*(*S*) positive. Only one isolate (MSL E.C 15) was positive both of *qnr*(*A*) and *qnr*(*S*) (Figure-2). A few studies have found bacteria harboring more than one *qnr* gene. This occurrence has been usually but not exclusively *qnr*(*S*) with either *qnr*(*B*) or *qnr*(*A*). Whether multiple Qnr proteins have an additive effect on the MIC is still unclear [24]. Stephenson *et al*. [9] reported 255 fluoroquinolone-resistant Enterobacteriaceae isolates in Jamaica; the sole *qnr*(*A*), *qnr*(*B*), and *qnr*(*S*) gene locus was identified in 48%, 1%, and 10% of the isolates, respectively. Another 36% of the isolates were positive for *qnr*(*A*) and *qnr*(*S*), and two isolates (both *E. coli*) had all three determinants. Previous studies in Czech Republic, plasmid-mediated resistance *qnr*(*B*) and *qnr*(*S*) genes were detected in 19% and 52% of the tested samples in 86 *E. coli* strains isolated from poultry as compared with other European countries [8]. Rodriguez *et al*. [25] reported that *qnr*(*A*)- and *qnr*(*B*)-positive plasmids often harbor genes that confer resistance to β-lactams, aminoglycosides, chloramphenicol, TE, sulfonamides, trimethoprim, and rifampin. The presence of *qnr* genes also increased fluoroquinolone MICs. Bacteria with plasmid-mediated quinolone resistance genes are not always resistant to fluoroquinolones due to the acquisition of low-level quinolone resistance. However, it is thought that when plasmids harboring *qnr* genes are transferred to various bacterial species, resistance to fluoroquinolone rapidly increases [26]. In Indonesia, there are no data about plasmid-mediated quinolone resistance in *E. coli* strains isolated from animals, despite few studies in human.

## Conclusion

The widespread use of antimicrobial agents caused an increase in the incidence of resistance in both pathogenic and endogenous bacteria, highlighting a serious health problem to human medicine. TE and quinolone antibiotics are commonly used in poultry, but overuse and misuse of this antibiotics may lead to an increased antimicrobial resistance. The emergence of antibiotic resistance cannot be separated from the existence of resistant gene. This present study revealed a high rate of resistance TE and quinolone antibiotics in *E. coli* isolates from clinical cases of avian colibacillosis in Sukabumi, Indonesia. The presence of *tet*(*A*) gene is more dominant than the *tet*(*B*) gene that encodes resistance to TE. As well as, *tet*(*A*) gene and *qnr*(*A*) were higher than *qnr*(*S*) and *qnr*(*B*) that encodes resistance to quinolone. Both of these resistance genes suggestive of potential horizontal transfer of the resistance genes between strains.

## Author’s Contributions

RSK designed the study and drafted the manuscript under the supervision of AI and NLPIM. AP collected samples and compiled the resource materials. RSK and AP performed the test and data analysis under the supervision of AI. RSK conducted molecular detection of resistance gene by PCR under the supervision of NLPIM. All authors have read and approved the final manuscript.
